# Maternal personality and postpartum mental disorders in Japan: the Tohoku Medical Megabank Project Birth and Three-Generation Cohort Study

**DOI:** 10.1038/s41598-022-09944-w

**Published:** 2022-04-16

**Authors:** Keiko Murakami, Mami Ishikuro, Taku Obara, Fumihiko Ueno, Aoi Noda, Tomomi Onuma, Fumiko Matsuzaki, Saya Kikuchi, Natsuko Kobayashi, Hirotaka Hamada, Noriyuki Iwama, Hirohito Metoki, Masatoshi Saito, Junichi Sugawara, Hiroaki Tomita, Nobuo Yaegashi, Shinichi Kuriyama

**Affiliations:** 1 Tohoku Medical Megabank Organization, Tohoku University, 2-1 Seiryo-machi, Aoba-ku, Sendai, Miyagi 980-8573 Japan; 2 Graduate School of Medicine, Tohoku University, 2-1 Seiryo-machi, Aoba-ku, Sendai, Miyagi 980-8575 Japan; 3 Tohoku University Hospital, 1-1 Seiryo-machi, Aoba-ku, Sendai, Miyagi 980-8574 Japan; 4 Faculty of Medicine, Tohoku Medical and Pharmaceutical University, 1-15-1 Fukumuro, Miyagino-ku, Sendai, Miyagi 983-8536 Japan; 5 International Research Institute of Disaster Science, Tohoku University, 2-1 Seiryo-machi, Aoba-ku, Sendai, Miyagi 980-8573 Japan

**Keywords:** Risk factors, Signs and symptoms

## Abstract

Personality has been shown to predict postpartum depressive symptoms (PDS) assessed by the Edinburgh Postnatal Depression Scale (EPDS). However, existing studies have not considered the underlying symptom dimensions in the EPDS. We analyzed data from 15,012 women who participated in the Tohoku Medical Megabank Project Birth and Three-Generation Cohort Study. Personality was assessed in middle pregnancy using the short-form Eysenck Personality Questionnaire-Revised. PDS were defined as EPDS score ≥ 9 at 1 month after delivery. The EPDS items were further divided into three dimensions: depressed mood, anxiety, and anhedonia. Multiple analyses were conducted to examine the associations of each personality scale with PDS and three dimensions in the EPDS, adjusting for age, parity, mode of delivery, education, income, and social isolation. The prevalence of PDS assessed by the EPDS at 1 month after delivery was 13.1%. Higher neuroticism scores were associated with PDS (odds ratio [OR], 2.63; 95% confidence interval [CI], 2.48 to 2.79) and all three dimensions (all *p* < 0.001). Lower extraversion scores were associated with PDS (OR, 0.74; 95% CI, 0.70 to 0.78) and all three dimensions (all *p* < 0.001). Lower psychoticism scores were associated with PDS (OR, 0.89; 95% CI, 0.85 to 0.94) and anxiety (*p* < 0.001), but not with depressed mood (*p* = 0.20) or anhedonia (*p* = 0.92). In conclusion, higher neuroticism and lower extraversion were associated with PDS and the three underlying dimensions in the EPDS, while lower psychoticism was associated with anxiety, but not with depressed mood or anhedonia.

## Introduction

Maternal mental disorders in the postpartum period are considered a major public health concern worldwide because of their adverse effects on the mother, child, and family^1^. Postpartum depression (PPD), a major postpartum mental disorder, creates personal suffering and diminishes the ability to function effectively in many spheres of maternal life^2^. Postpartum mental disorders can also interfere with the quality of parenting and increase the risks of psychological and developmental disturbances in children^3^. Therefore, early detection and preventive interventions for high-risk mothers are essential for both maternal and child health.

Research on postpartum mental disorders has largely focused on PPD^1^. Although the etiology of PPD is considered multifactorial, personality—individual differences in characteristic patterns of thinking, feeling, and behaving—is among the important characteristics that have been hypothesized to predict PPD^1,2^. Higher scores for neuroticism and lower scores for extraversion have consistently been shown to be associated with increased risk of PPD, while a limited number of studies have examined other personality traits such as openness, agreeableness, and conscientiousness and reported mixed findings^4–9^. There is limited evidence on the association between psychoticism and PPD^10,11^. The majority of these studies used the Edinburgh Postnatal Depression Scale (EPDS), which is designed to screen for postpartum depressive symptoms (PDS) specifically in the context of PPD^12^. There is now sufficient evidence that the EPDS is not unidimensional; examinations of the factor structure have consistently suggested that the EPDS incorporates an anxiety component^13–18^. Meanwhile, several studies have indicated a possible third underlying dimension in the EPDS for anhedonia^13–15,17,18^. Women scoring high in one dimension may not score high in the total EPDS score and consequently may not be flagged if the total EPDS score is used on its own. This means that examining the association between multiple personality traits and multiple EPDS dimensions can help to clarify the association between personality and psychopathology of PDS. To the best of our knowledge, no studies have examined the associations between personality and the different EPDS dimensions.

Considering the above circumstances, we aimed to examine the associations of maternal personality with PDS assessed by the EPDS and its three underlying symptom dimensions (depressed mood, anxiety, and anhedonia). As personality scales, we used neuroticism, extraversion, and psychoticism, the three major traits of personality proposed by Eysenck^19^, along with a lie scale to detect socially desirable responding as a validity scale.

## Methods

### Study population

**Figure 1:**
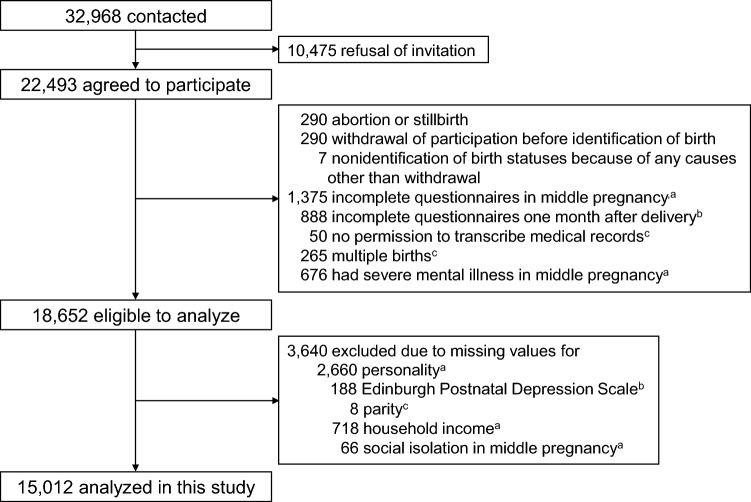
Flow diagram for the study participants in the present analysis of the Tohoku Medical Megabank Project Birth and Three-Generation Cohort Study. ^a^Data on personality, household income, and social isolation were obtained using questionnaires administered in middle pregnancy. ^b^Data on the Edinburgh Postnatal Depression Scale were obtained using questionnaires administered one month after delivery. ^c^Data on multiple births and parity were obtained transcribing medical records from obstetric clinics or hospitals.

Data were obtained from the Tohoku Medical Megabank Project Birth and Three-Generation Cohort Study (TMM BirThree Cohort Study), which was described elsewhere^20–23^. Pregnant women and their family members were contacted in approximately 50 obstetric clinics or hospitals in Miyagi Prefecture from 2013 to 2017. Of 32,968 pregnant women who were contacted, 22,493 agreed to participate. Among them, 3841 women were excluded owing to abortion or stillbirth, nonidentification of birth statuses, incomplete questionnaires, no permission to transcribe medical records, multiple births, and severe mental illness in middle pregnancy (14–27 weeks of gestation). Severe mental illness was defined as a score of ≥ 13 in the Japanese version of the K6 scale, which consists of six items for assessment of depressive mood and anxiety during the past 30 days (total score range: 0–24)^24–26^. Of the remaining 18,652 women, 3640 were excluded because of missing values for personality, EPDS, parity, household income, or social isolation during pregnancy. The remaining 15,012 women were included in the present study. Figure 1 shows a flow diagram for the study participants. We confirm that all procedures contributing to this work comply with the ethical standards of the relevant national and institutional committees on research involving human participants and with the Helsinki Declaration of 1975, as revised in 2008.

### Measures

Personality was assessed in middle pregnancy using the Japanese version of the short-form Eysenck Personality Questionnaire-Revised (EPQ-R)^27^. The short-form EPQ-R has 48 questions with dichotomized responses (yes or no), and there are 12 questions for each of four personality scales (neuroticism, extraversion, psychoticism, and lie)^28,29^. The neuroticism scale represents emotional instability and anxiousness, the extraversion scale represents sociability, liveliness, and surgency, and the psychoticism scale represents tough-mindedness, aggressiveness, coldness, and egocentricity^28,29^. The lie scale captures the extent to which the individual is “faking good,” due to unsophisticated dissimulation and social naivety or religiosity^28,29^. Scores for each scale range from 0 to 12, with higher scores indicating a greater tendency to possess the personality trait represented by each scale. Cronbach’s alpha coefficients were 0.79 for neuroticism, 0.81 for extraversion, 0.35 for psychoticism, and 0.65 for lie.

At 1 month after delivery, participants responded to the Japanese version of the EPDS^12,30^. The timing of 1 month after delivery overlaps with a medical examination and home visit service in Japan; therefore, it is easy for healthcare professionals to interact with both mother and infant at this time. The EPDS consists of 10 items assessing any symptoms of depression in the past 7 days: (1) laughing, (2) enjoyment, (3) self-blame, (4) anxiety, (5) scaredness, (6) finding it hard to cope, (7) finding it hard to sleep, (8) sadness, (9) crying, and (10) self-harm. Each item has four possible responses (0 to 3), with total scores ranging from 0 to 30. The Japanese version of the EPDS had good internal consistency (Cronbach’s alpha = 0.78) and test–retest reliability (rank correlation coefficient = 0.92)^30^. A score of ≥ 9 has been used as a cut-off score to screen for clinical diagnosis of PPD in Japan, with sensitivity of 75% and 82% and specificity of 93 and 95%^30,31^, and is considered to indicate experience of PDS. The Japanese version of the EPDS probably has a three-factor structure that includes depressed mood, anxiety, and anhedonia^13,15,32^. A large-scale nationwide birth cohort study in Japan revealed that a three-factor structure comprising depressed mood (items 7, 9, and 10), anxiety (items 3, 4, 5, and 6), and anhedonia (items 1 and 2) showed acceptably high goodness-of-fit, sufficient explanation of total variance, and good internal reliability^15^. Because there are no established cut-off values for the factor scores, we treated the scores of these three dimensions as continuous variables.

As covariates, we chose age, parity, mode of delivery, educational attainment, household income, and social isolation during pregnancy^33^. Age at delivery, parity and mode of delivery (cesarean section, others) were ascertained from participants’ medical records. Parity was dichotomized into nulliparous and multiparous. Educational attainment was divided into three categories: high school or lower (elementary, junior high school, or senior high school), college (2-year college or special training school), and university or higher (university or graduate school). There were many missing values partly because the data were collected at 1 year after delivery. Therefore, a “missing” category was created in addition to the above categories. Information on household income and social isolation was obtained from the questionnaire in middle pregnancy. Household income was divided into three categories: ≤ 3.99, 4.00–5.99, and ≥ 6.00 million Japanese yen. Social isolation was defined as a score of < 12 for the Japanese version of the abbreviated Lubben Social Network Scale^34,35^.

### Statistical analysis

The characteristics of the participants without PDS (EPDS score ≤ 8) and with PDS (EPDS score ≥ 9) were compared using Student’s *t*-test for continuous variables and the chi-square test for categorical variables. Multiple logistic regression analyses were conducted to examine the association between each personality scale and PDS. The odds ratio (OR) and 95% confidence interval (CI) were calculated for 1 standard deviation increase in each personality scale adjusted for covariates. Multiple linear regression analyses were conducted to examine the association between each personality scale and each EPDS dimension as follows: depressed mood, anxiety, and anhedonia. Regression coefficient (*β*) and 95% CI were calculated for 1 standard deviation increase in each personality scale adjusted for covariates.

All analyses were performed using SAS version 9.4 (SAS Institute Inc., Cary, NC). For all analyses, a two-tailed *p*-value < 0.05 was considered statistically significant.

### Ethics approval and consent to participate

The TMM BirThree Cohort Study protocol was reviewed and approved by the Ethics Committee of Tohoku University Tohoku Medical Megabank Organization (2013-1-103-1). The Tohoku University Tohoku Medical Megabank Organization established seven community support centers in Miyagi Prefecture as local facilities for voluntary admission-type recruitment and health assessment of participants^23^. Trained genome medical research coordinators were placed in each clinic, hospital, or community support center to provide information on the TMM BirThree Cohort Study to potential participants and to receive signed informed consent forms from the enrolled participants.

## Results

Table 1 shows the characteristics of the study participants. The prevalence of PDS assessed by the EPDS was 13.1%. Women with PDS were more likely to be younger, be nulliparous, have lower household income, and be socially isolated during pregnancy than women without PDS.

**Table 1:** Characteristics of participants: the Tohoku Medical Megabank Project Birth and Three-Generation Cohort Study.

	Total (*N* = 15,012)		Total EPDS score^a^				*p*-value^b^
			Non-PDS (*n* = 13,046)		PDS (*n* = 1966)		
Age (years), mean (SD)	31.9 (4.9)		32.0 (4.8)		31.1 (5.0)		< 0.001
Nulliparous, *n* (%)	7257 (48.3)		5973 (45.8)		1284 (65.3)		< 0.001
Cesarean section, n (%)	3465 (23.1)		3008 (23.1)		457 (23.3)		0.85
**Educational attainment, *n* (%)**							0.0014
High school or lower	3172 (21.1)		2728 (20.9)		444 (22.6)		
College	3847 (25.6)		3373 (25.9)		474 (24.1)		
University or higher	2919 (19.5)		2585 (19.8)		334 (17.0)		
Missing	5074 (33.8)		4360 (33.4)		714 (36.3)		
**Household income, *n* (%)**							< 0.001
≤ 3.99 million Japanese yen	5346 (35.6)		4490 (34.4)		856 (43.5)		
4.00–5.99 million Japanese yen	4914 (32.7)		4263 (32.7)		651 (33.1)		
≥ 6.00 million Japanese yen	4752 (31.7)		4293 (32.9)		459 (23.4)		
Social isolation during pregnancy, *n* (%)	2892 (19.3)		2313 (17.7)		579 (29.5)		< 0.001
**Dimensions in the EPDS, mean (SD)**							
Depressed mood	0.30 (0.85)		0.078 (0.33)		1.73 (1.57)		< 0.001
Anxiety	4.10 (2.26)		3.58 (1.88)		7.55 (1.37)		< 0.001
Anhedonia	0.18 (0.54)		0.069 (0.31)		0.89 (1.01)		< 0.001
**Subscales of the short-form EPQ-R, mean (SD)**							
Neuroticism score	5.79 (3.02)		5.44 (2.92)		8.10 (2.62)		< 0.001
Extraversion score	5.86 (3.28)		6.01 (3.26)		4.86 (3.19)		< 0.001
Psychoticism score	2.41 (1.57)		2.43 (1.57)		2.27 (1.58)		< 0.001
Lie score	5.28 (2.37)		5.36 (2.36)		4.74 (2.36)		< 0.001

EPDS, Edinburgh Postpartum Depression Scale; EPQ-R, Eysenck Personality Questionnaire-Revised; PDS, postpartum depressive symptoms; SD, standard deviation.

^a^Total EPDS score was dichotomized into non-PDS (score ≤ 8) and PDS (score ≥ 9).

^b^Obtained using Student's *t*-test for continuous variables and the chi-square test for categorical variables, comparing non-PDS and PDS groups.

Table 2 presents the associations between personality scales and PDS assessed by the EPDS. Higher neuroticism scores were associated with an increased risk of PDS; the multivariate-adjusted OR was 2.63 (95% CI, 2.48 to 2.79). Higher extraversion scores were associated with a decreased risk of PDS; the multivariate-adjusted OR was 0.74 (95% CI, 0.70 to 0.78). Higher psychoticism scores were associated with a decreased risk of PDS; the multivariate-adjusted OR was 0.89 (95% CI, 0.85 to 0.94). Higher lie scores were associated with a decreased risk of PDS; the multivariate-adjusted OR was 0.78 (95% CI, 0.75 to 0.82).

**Table 2:** Odds ratios (ORs) and 95% confidence intervals (CIs) for postpartum depressive symptoms for 1 standard deviation increase in each personality scale.

	Age-adjusted			Multivariate-adjusted^a^		
	OR (95% CI)		*p*-value	OR (95% CI)		*p*-value
Neuroticism	2.70 (2.55, 2.86)		< 0.001	2.63 (2.48, 2.79)		< 0.001
Extraversion	0.68 (0.65, 0.72)		< 0.001	0.74 (0.70, 0.78)		< 0.001
Psychoticism	0.91 (0.86, 0.95)		< 0.001	0.89 (0.85, 0.94)		< 0.001
Lie	0.78 (0.74, 0.82)		< 0.001	0.78 (0.75, 0.82)		< 0.001

Postpartum depressive symptoms were defined as a total score of the Japanese version of Edinburgh Postpartum Depression Scale ≥ 9.

^a^Adjusted for age at delivery, parity, mode of delivery, educational attainment, household income, and social isolation during pregnancy.

Table 3 presents the associations between personality scales and underlying symptom dimension in the EPDS. Higher neuroticism scores were associated with an increased risk of depressed mood (*β*, 0.20; 95% CI, 0.19 to 0.22), anxiety (*β*, 0.91; 95% CI, 0.88 to 0.94), and anhedonia (*β*, 0.087; 95% CI, 0.079 to 0.096). Higher extraversion scores were associated with a decreased risk of depressed mood (*β*, − 0.056; 95% CI, − 0.070 to –0.042), anxiety (*β*, − 0.31; 95% CI, –0.34 to –0.27), and anhedonia (*β*, − 0.045; 95% CI, − 0.054 to − 0.037). Higher psychoticism scores were associated with a decreased risk of anxiety (β, − 0.25; 95% CI, − 0.28 to − 0.22), but not with depressed mood (*β*, − 0.0088; 95% CI, − 0.022 to 0.0048) or anhedonia (*β*, − 0.00044; 95% CI, − 0.0090 to 0.0082). Higher lie scores were associated with a decreased risk of depressed mood (*β*, − 0.055; 95% CI, − 0.0068 to − 0.041), anxiety (*β*, − 0.30; 95% CI, − 0.34 to − 0.27), and anhedonia (*β*, − 0.021; 95% CI, − 0.030 to − 0.012).

**Table 3:** Regression coefficients and 95% confidence intervals (CIs) for each underlying symptom dimension in the EPDS for 1 standard deviation increase in each personality scale.

	Depressed mood			Anxiety			Anhedonia		
	*β*	(95% CI)^a^	*p*-value	*β*	(95% CI)^a^	*p*-value	*β*	(95% CI)^a^	*p*-value
Neuroticism	0.20	(0.19, 0.22)	< 0.001	0.91	(0.88, 0.94)	< 0.001	0.087	(0.079, 0.096)	< 0.001
Extraversion	− 0.056	(− 0.070, − 0.042)	< 0.001	− 0.31	(− 0.34, − 0.27)	< 0.001	− 0.045	(− 0.054, − 0.037)	< 0.001
Psychoticism	− 0.0088	(–0.022, 0.0048)	0.20	− 0.25	(− 0.28, − 0.22)	< 0.001	− 0.00044	(− 0.0090, 0.0082)	0.92
Lie	− 0.055	(− 0.068, − 0.041)	< 0.001	− 0.30	(− 0.34, − 0.27)	< 0.001	− 0.021	(− 0.030, − 0.012)	< 0.001

EPDS, Edinburgh Postpartum Depression Scale.

^a^Adjusted for age at delivery, parity, mode of delivery, educational attainment, household income, and social isolation during pregnancy.

## Discussion

The present study examined the associations of maternal personality with PDS assessed by the EPDS and its three dimensions (depressed mood, anxiety, and anhedonia) in Japan. Higher scores for neuroticism, lower scores for extraversion, and lower scores for psychoticism were associated with an increased risk of PDS. Higher scores for neuroticism and lower scores for extraversion were associated with increased risks of the three EPDS dimensions, while lower scores for psychoticism were associated with an increased risk of anxiety, but not with depressed mood or anhedonia. Higher lie scale scores were associated with decreased risks of PDS and the three EPDS dimensions.

The prevalence of PDS assessed by the EPDS at 1 month after delivery was 13.1%. A recent meta-analysis reported that the PDS prevalence at 1 month after delivery based on EPDS data was 14.1% among Japanese women^36^. The present study assessed a group of mothers without severe mental illness in middle pregnancy to avoid the possibility of distorting the personality assessment, because depressive symptoms are associated with changes in personality that may be temporary or persistent^37^. Although antenatal depression and anxiety are high risk factors for PDS^33^, the PDS prevalence assessed by the EPDS in the present study did not differ markedly from the prevalence among Japanese women, some of whom may have mental disorders during pregnancy. The present findings indicate that emphasis should also be placed on mothers without mental disorders during pregnancy.

Mothers with higher neuroticism scores had a higher risk of PDS assessed by the EPDS. This finding is consistent with meta-analyses that identified neuroticism as a risk factor with moderate to strong associations with PPD^2^. Longitudinal analyses adjusted for baseline depressive symptoms also showed that higher neuroticism was associated with an increased risk of depressive symptoms during follow-up in the general population^37^. Neuroticism is characterized by a tendency to experience fear, anger, and sadness and a susceptibility to the effects of stress on mood^38^. The transition to motherhood can be a stressful event, owing to emotional and physical changes as well as new responsibilities and demands^39^. Mothers with higher neuroticism scores may have less well-regulated emotional responses to this stressful event. The present study also showed that higher neuroticism scores were associated with an increased risk of anxiety dimension, as well as depressed mood dimension. This result is consistent with the concept that anxiety can be considered a lower-order facet of neuroticism ^40^.

Mothers with lower extraversion scores had a higher risk of PDS assessed by the EPDS. This finding is consistent with most studies on the association between extraversion and PDS^7–9^. Longitudinal analyses adjusted for baseline depressive symptoms also showed that lower extraversion was associated with an increased risk of depressive symptoms during follow-up in the general population^37^. Extraversion is characterized by a tendency to experience positive emotions, as well as to be gregarious and engaged with the environment^38^. Mothers with lower extraversion scores may take a negative attitude toward their new responsibilities as a mother. The present study also showed that lower extraversion scores were associated with an increased risk of anxiety dimension, as well as depressed mood dimension. There is only limited evidence on the association between personality and postpartum anxiety^8^, partly because mental health research and clinical practice have disproportionately targeted depression despite the high prevalence of postpartum anxiety^41^. Because anxiety has been a central component of theories for personality and psychopathology^40^, further understanding of the association between personality and postpartum anxiety is warranted.

Mothers with lower psychoticism scores had a higher risk of PDS assessed by the EPDS. There is only limited evidence on the association between psychoticism and PDS because the majority of the existing studies were based on the Five-Factor Model, which consists of neuroticism, extraversion, agreeableness, conscientiousness, and openness to experience^42^. It has been suggested that Eysenck’s psychoticism scale is equivalent to a blend of low conscientiousness and low agreeableness in the Five-Factor Model^42^. A cohort study in Spain showed that psychoticism did not predict PDS assessed by the EPDS at 8 and 32 weeks as well as a major depressive episode during 32 weeks after childbearing^11^. A case–control study among parous women in the United Kingdom found no differences in psychoticism between women with recurrent major depressive disorder who did or did not have a history of PPD^10^. The present study showed no association between psychoticism and depressive mood dimension in the EPDS. These results suggest that psychoticism is not associated with the depressive dimension of postpartum mental disorders. The present study also showed that lower psychoticism was associated with an increased risk of anxiety dimension in the EPDS. Anxiety is a negative affective state that occurs in response to a perceived threat and includes an attentional bias toward the potential threat, physiological responses including sympathetic nervous system activation, and action tendencies motivated to cope with the negative affect and/or threat cues^40^. Psychoticism is characterized by a temperamental disposition trait that renders a person more likely to succumb to a functional psychosis, given sufficient stress, resulting in a similar state to those in psychotic patients^28^. The dimension of psychoticism taps several facets such as hostility, cruelty, lack of empathy, and non-conformism^29^. These characteristics of the psychoticism scale may reduce postpartum anxiety.

Mothers with higher lie scale scores had a lower risk of PDS assessed by the EPDS. The lie scale was originally introduced into personality scales to capture the extent to which the individual is “faking good” for scores on other scales^28^. The simple interpretation of the lie scale as a validity scale is that women who pretend to be healthy are more likely to have lower EPDS scores, which may partially explain the association between higher lie scale scores and a lower risk of PDS assessed by the EPDS and its three dimensions observed in the present study. However, it has recently been suggested that this should be interpreted as measuring a personality dimension in its own right^43^. The lie scale may reflect social naivety, religiosity^27^, social acquiescence, or self-insight^44^, which may underlie “faking good.” These characteristics may also affect postpartum mental health.

The present findings have implications for preventing postpartum mental disorders. The postpartum period is a complex time with immense changes at the biological, psychological and social levels, and it is therefore difficult to differentiate which factors in each of these domains play the most significant roles in the onset of mental disorders at this time. However, the associations observed in the present study indicate that failure to account for personality may lead to inaccurate risk estimates of mental disorders during the postpartum period. Personality tests are noninvasive and can be conducted without special equipment or techniques; therefore, they can be easily introduced into clinical practice. Pregnancy may be an ideal period to conduct screening programs because women are in regular contact with healthcare professionals. Assessment of personality during pregnancy may be useful for screening in obstetric practice to perform early interventions for women at high risk of mental disorders, which may contribute to optimal allocation of prevention resources.

The present study has several limitations. First, the study was conducted in one of the 47 prefectures in Japan; therefore, generalizability is limited. Second, as in most epidemiological studies, maternal personality was self-reported. However, self-reported data are often preferred over observer ratings of personality because observers do not have access to a person’s internal motives, emotions, and thoughts, only to external behavioral manifestations of these factors. Furthermore, the reliability and validity of the Japanese version of the short-form EPQ-R have been verified^27^. Third, Cronbach’s alpha coefficient for psychoticism was low, similar to previous findings^27^. The low alpha values for psychoticism may be caused by the narrow range of scoring and the grossly skewed distribution^27^. Fourth, information on personality was obtained in middle pregnancy; the possibility cannot be ruled out that the obtained personality scores were affected by pregnancy. However, personality has been reported to be relatively stable in adulthood^45^ and personality assessed during pregnancy has been suggested to be associated with peripartum changes in psychopathological symptoms^46^. Therefore, personality may be a marker for each dimension of the EPDS, although unmeasured common factors during pregnancy may influence both personality and the EPDS, which may lead to the overestimation of the association between personality and PDS. Finally, a group of mothers with severe mental illness in middle pregnancy were excluded, although antenatal depression, antenatal anxiety, and previous depressive illness are the strongest PDS risk predictors^33^. However, analyses including mothers with severe mental illness in middle pregnancy produced similar results to those obtained in the present study.

## Conclusions

Maternal personality was associated with PPD assessed by the EPDS and its underlying symptom dimensions. Higher neuroticism scores and lower extraversion scores were associated with increased risks of PDS, depressed mood, anxiety, and anhedonia. Although lower psychoticism scores were associated with an increased risk of PDS, they were only associated with anxiety, and not with depressed mood or anhedonia. Higher lie scale scores were associated with decreased risks of PDS, depressed mood, anxiety, and anhedonia. Although the mechanisms underlying these associations remain unclear, assessment of personality may be an inexpensive early detection method for pregnant women at future risk for postpartum mental disorders. Our findings provide clues toward the design of more effective interventions for preventing postpartum mental disorders, with consequent improvements in maternal and child health.

## Data Availability

Data obtained through the TMM BirThree Cohort Study are incorporated into the TMM biobank. All data analyzed during the present study are available for research purpose with the approval by the Sample and Data Access Committee of the TMM biobank.

## Abbreviations

95% CI: 95% Confidence interval
EPDS: Edinburgh Postnatal Depression Scale
EPQ-R: Eysenck Personality Questionnaire-Revised
OR: Odds ratio
PDS: Postpartum depressive symptoms
PPD: Postpartum depression
TMM BirThree Cohort Study: Tohoku Medical Megabank Project Birth and Three-Generation Cohort Study
